# Non-invasive MRI of choroid plexus-cerebrospinal fluid water exchange using multi-TE FLAIR

**DOI:** 10.1162/IMAG.a.1097

**Published:** 2026-01-16

**Authors:** Shereen Nizari, Charith Perera, Lydiane Hirschler, Ian F. Harrison, Mark F. Lythgoe, Matthias J.P. van Osch, David L. Thomas, Jack A. Wells

**Affiliations:** UCL Centre for Advanced Biomedical Imaging, Division of Medicine, University College London, United Kingdom; C.J. Gorter MRI Center, Department of Radiology, Leiden University Medical Center, Leiden, Netherlands; Neuroradiological Academic Unit, Department of Translational Neuroscience and Stroke, UCL Queen Square Institute of Neurology, University College London, London, United Kingdom; Dementia Research Centre, UCL Queen Square Institute of Neurology, University College London, London, United Kingdom

**Keywords:** choroid plexus, blood-cerebrospinal fluid barrier, MRI, FLAIR, CSF, exchange, glymphatics

## Abstract

The choroid plexus (CP), or blood-cerebrospinal fluid barrier, performs unique and diverse roles in support of brain homeostasis. Novel and non-invasive imaging biomarkers of choroid plexus physiology may be useful to further our understanding of its role in the development of pathology. Here, we introduce the concept of measuring water exchange between the choroid plexus tissue and the proximal CSF using a multiple TE fluid-suppressed (FLAIR) acquisition. By fitting the MRI signal at the choroid plexus acquired with a multi-TE FLAIR readout to a two-compartment bi-exponential model, we observed that the slow decaying T2 component of the signal had a T2 highly similar to that of cerebrospinal fluid (CSF). This finding, in turn, provides evidence that, paradoxically, this signal derives from CSF in a FLAIR image. The specific spatial co-localization of this long-T2 signal to the CP within the lateral ventricles provides evidence that this reflects the exchange of water molecules between the CP tissue and the CSF during the inversion time. A reduction in choroid plexus-CSF water exchange rate was then detected in the aged vs. young mouse brain using this method. Preliminary application of the method to the human brain at 3T, however, yielded weaker results which suggest that the method may not be able to capture this phenomenon clinically with equivalent sensitivity. Nonetheless, in pre-clinical studies, this novel MRI contrast mechanism provides a specific and quantitative signature of choroid plexus physiology and thus may represent a useful non-invasive imaging biomarker of CP dysfunction.

## 1 Introduction

The choroid plexus (CP) floats within the cerebrospinal fluid (CSF) in particular locations within the brain’s lateral ventricles. The CP forms the blood-cerebrospinal fluid-barrier (BCSFB), a unique interface joining the systemic circulation and the central nervous system. The CP drives a vast array of physiological process in service of healthy brain function (Courtney et al., 2024; MacAulay et al., 2022). Perhaps its most important role is the secretion of CSF, delivering ~500 ml per day to the brain via the BCSFB (Spector et al., 2015). As such, optimal CP function may support the efficacy of CSF-mediated brain clearance processes thought to be important in preventing the accumulation of proteins that define common age-related neurogenerative conditions such as Alzheimer’s disease (Bedussi et al., 2015; Iliff et al., 2012). Of particular relevance to the MRI method introduced here is that, despite its relatively small size, the BCSFB has a large surface area interfacing the CSF, estimated to be approximately one-half that of the blood-brain-barrier, from measurements in the rat brain (Keep & Jones, 1990). Novel and non-invasive methods of assessing CP physiology are needed to better understand its role in disease and to fully realize the potential of the CP as a diagnostic and therapeutic target for neurological conditions such as Alzheimer’s disease and hydrocephalus.

Fluid attenuated inversion recovery (FLAIR) is a widely used MRI method that suppresses the signal from CSF by acquiring data at its null point following an inversion pulse. Here, we introduce the concept of measuring water exchange between the CP and CSF using a multi-echo FLAIR MRI sequence. We present evidence that this technique can capture rapid exchange of water between the CP and CSF during the inversion time of the FLAIR sequence with high sensitivity and specificity to the CP tissue in the mouse brain. This non-invasive method may, therefore, be sensitive to functional changes that occur within the choroid plexus with aging and disease such as altered microvilli behavior and transporter expression. Given the marked changes reported in CP structure and function in the aged brain, we applied the method to the aged mouse to investigate possible differences in estimated water exchange at the CP. Finally, in a pilot study we applied the method to the human brain at 3T.

This work presents evidence that multi-TE FLAIR is sensitive to the rate of water exchange between the CP and CSF. As such, this approach represents a novel, non-invasive, and semi-quantitative measurement of brain physiology that may represent a valuable non-invasive biomarker of CP function.

## 2 Methods

### 2.1 Background theory and pilot experiments

Accurate measurement of the T2 of the CP could provide a valuable biomarker of derangement to CP physiology in age and disease. To our knowledge, there is only one previously reported reliable measurement of the T2 of the CP tissue in the literature, where Lee and colleagues performed multi-TE measurements and found convincing evidence for two distinct T2 ‘peaks’ of ~40 ms (corresponding to the CP tissue) and ~300 ms (corresponding to the CSF) at 9.4T (Lee et al., 2022). Therefore, we thought it would be potentially advantageous to map the T2 of the CP tissue by using a multi-TE FLAIR sequence in order to null the CSF signal (based on the different T1s of CP tissue and CSF) and fit a mono-exponential model to the multi-TE data to estimate the T2 of the CP tissue. Based on the measures of Lee et al. (2022), we anticipated that the T2 of the CP using this approach (with the CSF signal suppressed) would be ~40 ms, similar to that of brain tissue at 9.4T.

We performed a pilot study and found, however, that using the FLAIR multi-TE sequence, that the T2 of the signal co-localised to the CP was markedly greater than expected based on the previous measurements by Lee et al. (and markedly greater than the T2 of the surrounding grey matter tissue in the mouse brain). This observation motivated us to perform additional experiments to understand the physiological underpinnings of this high T2 value. These experiments centred around bi-exponential fitting of the multi-TE FLAIR signal that was co-localised to the CP, in order to evaluate contributing components to the measurement. This then led us to hypothesize that exchange effects were significantly influencing the observed signal behaviour.

Following the IR pulse and the inversion time (TI), at the time of measurement, when we fitted the signal co-localised to the CP as a function of TE to a bi-exponential model, the long T2 component very closely matched the T2 of CSF (estimated from the identical acquisition and analysis but without the IR preparation). This suggested one of two scenarios: 1) there was a particular water compartment located in or proximal to the CP that had a T2 that matched the CSF but a markedly different T1 to the CSF; 2) rapid exchange of water was occurring during the TI between the CP (relatively short T1) and the proximal CSF (relatively long T1) which acted to markedly reduce the effective T1 of water in the CSF proximal to the CP at the time of the multi-TE readout (in other words, during the TI the fast exchange of water between the CP and proximal CSF meant that water molecules in the CSF proximal to the CP at the time of the multi-TE readout have spent time within the CP tissue which possesses a relatively short T1 compared to the CSF, and thus appeared visible in the FLAIR acquisition).

Here, we propose that the latter scenario is more likely as, for a given tissue-type, T1 and T2 tend to be closely correlated in-vivo. Hence, we propose that the multi-TE FLAIR approach provides a measure of water exchange rate between the CP tissue and local CSF.

#### 2.1.1 Pre-clinical MRI

All experiments were performed in accordance with the European Commission Directive 86/609/EEC (European Convention for the Protection of Vertebrate Animals Used for Experimental and Other Scientific Purposes) and the United Kingdom Home Office Animals (Scientific Procedures) Act (1986). All mice were acclimatized in an animal house, prior to data acquisition, with a 12-hour light/12-hour dark cycle with food and water provided ad libitum.

Images were acquired on a horizontal-bore 9.4T Bruker preclinical system (BioSpec 94/20 USR; Bruker) using a 440-mT/m gradient set with an outer and inner diameter of 205 mm and 116 mm, respectively (BioSpec B-GA 12S2), a 86-mm volume transit RF coil, and a four-channel receiver-array coil designed for the mouse brain (Bruker). All mice were induced with 2% isoflurane anaesthetic in a 4:1 mixture of room air and O_2_, adjusted to 1.5% to maintain the respiration rate at ~150 bpm, which was measured using a pressure pad and monitored throughout the scan. A rectal probe (SA Instruments, Stony Brook, NY) was used to monitor the core body temperature, which was maintained at 37.0 ± 0.5 °C via an adjustable water bath supplied to a mouse heating pad (Bruker BioSpec; Bruker, Kontich, Belgium). The multi-TE FLAIR sequence was taken from the Resource for Experimental Magnetic Resonance Microstructure Imaging toolbox by Mark Does and Kevin Harkins (https://remmi-toolbox.github.io/).

#### 2.1.2 Part i) Details of pilot experiment: multi-TE FLAIR to measure the T2 of the choroid plexus

A single male C57BL/6 mouse at 3 months of age was used in the pilot study. After acquisition of scout images, a 3D T2-weighed fast-spin-echo sequence was implemented in order to visualise the location of the CP tissue within the lateral ventricles, with the following parameters: matrix size: 196 × 196 × 24; Field of View (FOV): 19.6 × 19.6 × 2.4 mm; TR = 5s; TE = 176.16; echo train length = 64; echo spacing = 7.34 ms; number of averages = 1; and acquisition time = 6 minutes and 45 s.

The 3D T2-weighted images were used to position the multi-TE FLAIR acquisition with a single coronal slice centered on a section of the lateral ventricles where the choroid plexus is clearly visible (given the relatively high between-mouse variability in the location of the choroid plexus within the lateral ventricles). A multi-TE FLAIR (spin echo) acquisition was then run with the following parameters: brain-wide inversion pulse (thickness = 2.5 cm), slice thickness = 0.25 mm; TR = 6 s; TI = 2.15 s; matrix size = 96 × 96; FOV = 15 × 15 mm; first echo time = 6.74 ms; echo spacing = 4.96 ms; number of echoes = 24; and number of averages = 9. The inversion pulse was a slice selective pulse with a length of 1.7 ms and bandwidth of 2000 Hz (with a numerically optimized pulse shape using the Shinnar–Le Roux algorithm). The TI of 2.15s was chosen as visual inspection of the images indicated good nulling of CSF signal (with no CP tissue).

##### 2.1.2.1 Analysis

An ROI was drawn in the cortex, CP, lateral ventricle CSF (no CP tissue) and in the background (air) based on the FLAIR image at the shortest TE. Example ROIs for these regions are shown in Supplementary Figure S1. The mean signal within each of the respective ROIs was taken, and the apparent T2 (T2_app_) was calculated by fitting the signal within the CP and cortex ROIs to a simple mono-exponential model (i.e., signal (TE) = S0 × exp (-TE/ T2_app_). To generate T2_app_ maps, the images were first thresholded so that voxels with an SNR less than 15 × the standard deviation of the background were not fitted and their value was assigned as 0.

#### 2.1.3 Part ii) Performing bi-exponential fitting to understand more about the underpinnings of the long T2 component at the CP in the multi-TE FLAIR acquisition (n = 6)

Based on the results of the pilot study (higher than expected measured CP T2_app_ values; see Section 3), the following experiments were designed to address the following hypotheses:

1. Is the decay of the signal co-localized to the CP from the multi-TE FLAIR acquisition well described by a biexponential model and (ii) if so, what are the T2 values for each component?
  ..and after finding evidence that the long T2 component matched the T2 value of the CSF:
2. Does the spatial localization of this putative CSF signal overlay with the spatial location of the choroid plexus within the lateral ventricles?

In order to investigate the above hypotheses, we performed the following acquisitions:

Three male and three female C57BL/6 mice (~3 months of age) were used for these experiments. The same high-resolution 3D T2-weighed fast-spin-echo sequence was implemented as described above. A multi-TE FLAIR (spin echo) acquisition (with the coronal slice centred where the CP is visible within the lateral ventricles) was then acquired with the following parameters: slice thickness: 0.35 mm; TR= 6 s; TI = 2.15 s; matrix size = 96 × 96; FOV = 15 × 15 mm; first echo time = 6.74 ms; echo spacing = 4.96 ms; number of echoes = 48; number of averages = 9; and inversion pulse width = 35 mm (to cover entire mouse brain). Here, the number of echoes was increased from 24 to 48 in order to improve the precision of a bi-exponential fit to the signal as a function of TE. An identical scan was then run but without the FLAIR inversion pulse so as not to suppress CSF signal in this acquisition.

##### 2.1.3.1 Analysis

Within the multi-TE FLAIR sequence, an ROI was drawn within the choroid plexus (taking particular care not to overlap with brain tissue at the ependymal wall) and the mean signal was taken for each TE. This signal was then fit to an unconstrained 4-parameter bi-exponential model (assuming no exchange between compartments during the TE):

$$\begin{array}{l} \text{Signal}=\text{S}0_{\text{short T}2}\times \text{exp}(-\text{TE}/\text{T}2_{\text{short}})\\ +\text{S}0_{\text{long T}2}\times \text{exp}(-\text{TE}/\text{T}2_{\text{long}}) \end{array}$$ (1)

where S0_short T2_ is the signal contribution at TE = 0 of the relatively short T2 component (T2_short_) and S0_long T2_ is the signal contribution at TE = 0 of the relatively long T2 component (T2_long_). The Akaike Information Criteria was calculated for both a mono-exponential model and the bi-exponential model using the ‘AIC’ function in Matlab (R2022a). The percentage contribution of the S0_long T2_ component to the total measured signal was calculated using the following: 100 × (S0_long T2_/[S0_long T2_ + S0_short T2_]).

The same procedure (using the identical ROI) was applied to the multi-TE data acquired without the FLAIR IR pulse where we surmised that the calculated T2_long_ (FLAIR_OFF_) would yield an accurate estimate of the T2 of the CSF given the marked CSF contributions to these images without the FLAIR IR preparation (as is evident in Fig. 2, for example). Here, we hypothesized that if the estimated T2_long_ (FLAIR_ON_) matched well with the estimated T2_long_ (FLAIR_OFF_—an accurate estimate of the T2 of the CSF) then this would provide evidence that there is a marked signal contribution from the CSF to the signal co-localised to the CP from the multi-TE FLAIR acquisition.

To investigate if S0_long T2_ from the multi-TE FLAIR acquisition is specifically spatially co-localized to the CP, we then generated voxel-wise maps of S0_long T2_ by fitting the multi-TE FLAIR data to the same biexponential model above but with T2_long_ and T2_short_ fixed at 163 ms and 33 ms, based on the mean T2_long_ and T2_short_ estimates calculated by the 4-parameter unconstrained fit on the CP ROI (described above). For the generation of the maps, the images underwent thresholding so as not to fit voxels that have an SNR less than 15 based on the FLAIR image at the earliest TE.

Within these maps, an ROI was drawn at the ependymal wall of the ventricles and the mean S0_long T2_ /[S0_short T2_ + S0_long T2_] ratio (henceforth called S0_CSF_/[S0_CP_ + S0_CSF_] based on the findings from the unconstrained biexponential fit described above) was taken.

#### 2.1.4 Part iii) Investigating the effects of inversion pulse width on the multi-TE FLAIR measurements

The spatial width of the inversion pulse used in the FLAIR preparation for the previous measurements was designed to invert all of the CSF in the brain, in order to prevent CSF inflow artifacts. However, we have previously shown that it is possible to measure the CP-mediated delivery of water from the blood into the CSF using a flow-sensitive alternating inversion recovery arterial spin labeling method with a long TE (coined BCSFB-ASL (Evans et al., 2020)). It is possible, therefore, that the multi-TE FLAIR measurements performed so far, may primarily reflect a similar physiological process of blood-water delivery. To investigate this, here we compared multi-TE FLAIR measurements with the previously used brain-wide inversion pulse width of 3 cm with a global inversion across the entirety of the body of the mouse (which will also affect the longitudinal magnetisation of the inflowing blood water). However, because of the high signal contributions of the long T2 component recorded in part ii) relative to the relatively small signal changes (tagged-control) that form the basis of the BCSBF ASL measurement (Evans et al., 2020), here we hypothesized that a different physiological phenomenon is at play (exchange of water molecules between the CP tissue and CSF during the TI) and therefore changing the width of the inversion pulse in this way would have minimal effect on the S0_CSF_/[S0_CP_ + S0_CSF_] measurements at the CP.

Five male C57BL/6 mice were used in these experiments. The same MRI sequence as described in part ii) was acquired but with 3 averages. The identical sequence was then run but with a global inversion across the body of the mouse.

##### 2.1.4.1 Analysis

An ROI across the CP was then drawn based on the FLAIR image at the earliest TE, and the same ROI was used to extract the signal from the CP across both acquisitions (with different inversion pulse widths). The respective signal was then fitted to the same 4 parameter unconstrained bi-exponential model (Eq. 1), and S0_CSF_/[S0_CP_ + S0_CSF_] was calculated as a measure of CP-CSF water exchange rate.

#### 2.1.5 Part iv) Application to the aged mouse brain

MRI experiments were performed on male C57BL/6 mice at 6 and 24 months of age (Janvier labs) (n = 10 and n = 12 respectively). In these mice, an initial series of scans were run in order to optimize the TI for effective nulling of CSF. This was important as possible systematic differences in the T1 of the CSF between the young an aged cohorts may have resulted in a systematic difference in the estimated S0_long T2_ if TI was kept constant. This was achieved by converting the multi-TE FLAIR sequence into an FSE sequence at a single effective echo time with an identical echo train length (48) and echo spacing (4.96 ms) meaning that the timings of the RF pulses within the sequence were identical. Separate images were acquired using this sequence at TIs of 1700, 1900, 2300, and 2500 ms in order to estimate the null point of CSF (with the CSF signal at TI = 1700 and 1900 ms before the null point and the CSF signal at TI = 2300 and 2500 ms after the null point). Within these images an ROI was drawn within the ventricular CSF (no CP tissue) and the mean signal as a function of TI was fitted to a simple linear function as an approximation to the shape of the inversion recovery plot near the null point of the curve. From this fit, the TI corresponding to the null point of the CSF was calculated and used as the TI for the final multi-TE FLAIR acquisition. The same multi-TE FLAIR sequence was then acquired as in part ii).

##### 2.1.5.1 Analysis

An ROI was drawn in the CP based on the multi-TE FLAIR image at the shortest TE, and the mean signal was then fitted to a 4 parameter bi-exponential model (Equation 1). The T2_Long_ was constrained to have a maximum value of the mean of the calculated T2_Long_ + two standard deviations and a minimum value of the mean of T2_Long_—two standard deviations (based on the results from the n = 6 mice in part ii)). The T2_short_ was constrained in an identical way.

Here, we hypothesized that the ratio of CSF signal to CP signal to (S0_CSF_/[S0_CP_ + S0_CSF_]_)_ would be reduced in the old mice relative to the young as the degree of water exchange rate between the CSF and CP tissue is reduced. A standard 2-tailed t-test was used to investigate possible difference between the (S0_CSF_/[S0_CP_ + S0_CSF_]_)_ ratios between the aged and young cohort.

#### 2.1.6 Part v) preliminary application of the multi-TE FLAIR measurement to the human brain at 3T

Two healthy volunteers were imaged using two 3T MRI scanners: a Siemens MAGNETOM PrismaFit (at the National Hospital for Neurology and Neurosurgery, Queen Square, London, UK) and a Philips Achieva scanner (in Leiden University Medical Center).

##### 2.1.6.1 Volunteer 1: Siemens 3T

A single healthy male volunteer (39 years of age) was scanned after providing informed consent conforming to local ethical regulations. Imaging data were acquired using a 64-channel head and neck array receiver coil and a body transmit coil. A vendor-provided 2D multi-TE CPMG sequence was acquired with and without FLAIR preparation. A single axial slice (thickness = 5 mm) was manually positioned to include both the rostral (no CP) and caudal (CP) section of the lateral ventricles. Images were acquired at 32 echo times ranging from 50 ms to 1600 ms, with an echo spacing of 50 ms. The matrix size was 256 × 256, FOV = 25.6 cm × 25.6 cm (in plane voxel size 1 mm × 1 mm), slice selective inversion and imaging pulses were used; TR = 4800 ms; TI = 1400 ms (chosen by acquiring lower resolution images at long TE over a range of TI values and identifying null point of ventricular CSF).

##### 2.1.6.2 Volunteer 2: Philips 3T

A single healthy male volunteer (35 years of age) was scanned at the LUMC after providing informed consent in accordance with the Leiden University Medical Center Institutional Review Board (Leiden, the Netherlands). A body transmit coil was used along with a commercial 32-channel receive head-coil to perform the imaging. A vendor providing multi-TE 2D-FLAIR sequence with a turbo-spin echo readout was used. Similar to the measurement in volunteer 1, a single axial slice (thickness 5 mm) was manually positioned on the lateral ventricles to include both the rostral (no CP) and caudal (CP) sections of the ventricle. Images were acquired at 32 echo times ranging from 40 ms to 1280 ms with an echo spacing of 40 ms using the following parameters: TR = 4800 ms; TI = 1450 ms; FOV = 25.6 × 25.6 cm (in plane voxel size 1 mm x 1 mm); and matrix size of 256 × 256. A non-selective inversion pulse was used for the FLAIR preparation, and flow compensation was applied through-plane. Similarly to the acquisition on the Siemens system, the TI was carefully chosen based on pre-scans where TI was varied at long TE images that were used to assess the degree of CSF nulling in the ventricles.

##### 2.1.6.3 Analysis

For both imaging data sets, ROIS were drawn in the cortex, the background (air), and in the rostral (no CP) and caudal sections (CP) of the lateral ventricles and the mean signal was plotted a function of TE.

## 3 Results

### 3.1 Part i) The pilot study returned high T2 values at the choroid plexus using a multi-TE FLAIR acquisition

**Fig. 1.**
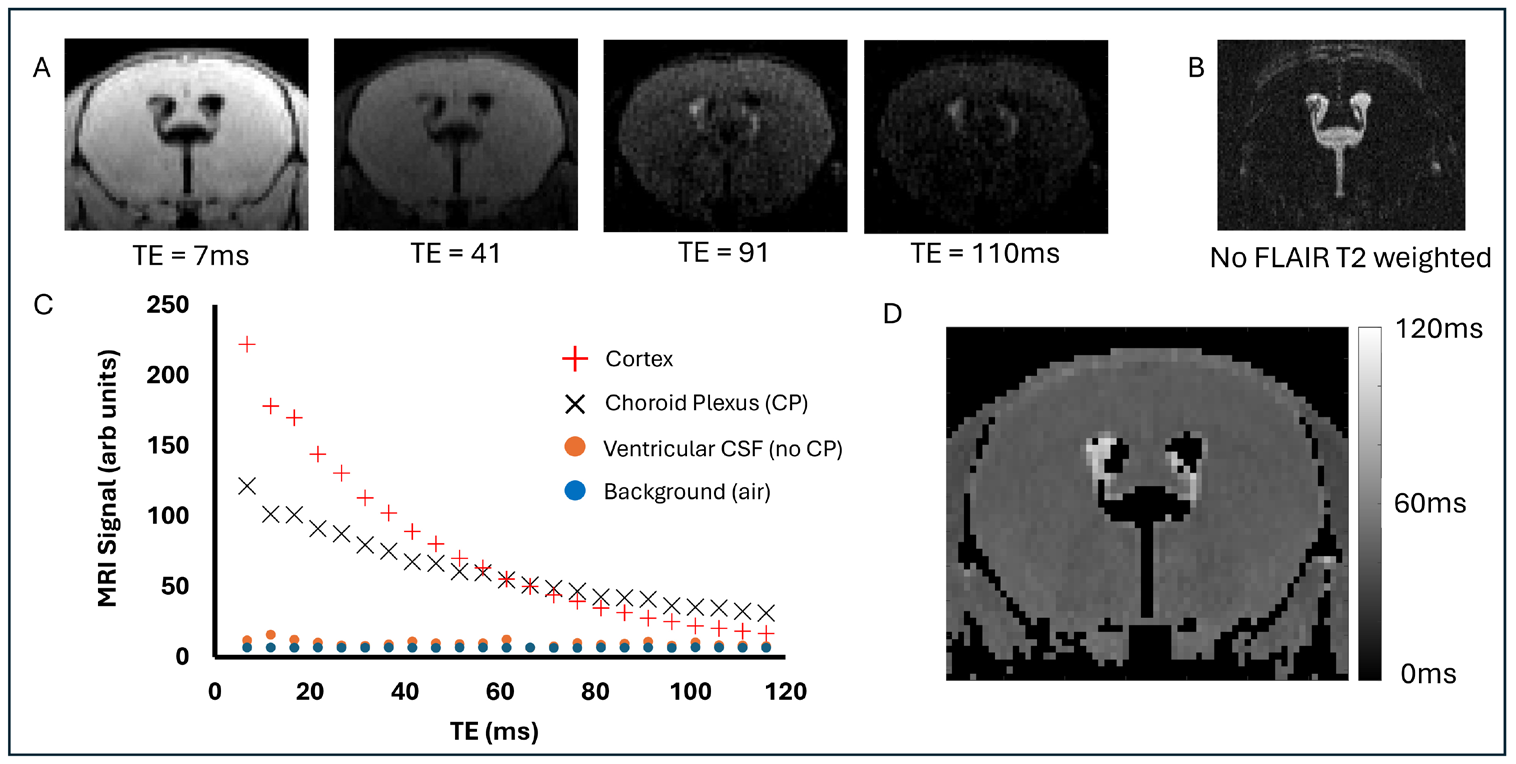
Pilot Study Returned High T2 Values at the Choroid Plexus using Multi-TE FLAIR. (A) Selected coronal (approx. -0.3 mm bregma) FLAIR images captured at increasing TE. (B) Coronal high resolution T2 weighted image to visualise the location of the choroid plexus. (C) Mean MRI signal as a function of TE from four regions of interest indicated in the insert. (D) T2_app_ map generated from the multi-TE FLAIR acquisition.

Figure 1A shows example FLAIR images acquired at increasing TE. As expected, the location of the CP within the lateral ventricles can be seen in the FLAIR image at the earliest TE (7 ms) which, from visual inspection, matches well with the high-resolution T2-weighted images (Fig. 1B - a separate high resolution scan designed to visualise the location of the CP within the lateral ventricles). As TE increases, the CP appears relatively bright compared to the surrounding parenchymal tissue (Fig. 1A), implying that the signal at the CP has a relatively long apparent T2 (T2_app_) compared to other parenchymal brain tissue (e.g., the cortex or striatum). Taking the mean MRI signal from an ROI co-localized to the CP (Fig. 1C) showed a relatively slow rate of transverse decay compared to cortical tissue, returning T2_app_ values of 84 ms and 42 ms respectively. The signal taken from an ROI within the ventricles (but not containing CP tissue) was close to that of the background noise, suggesting good suppression of ventricular CSF (not directly proximal to the CP) by the FLAIR preparation, as intended (Fig. 1C). The signal at the earliest TE for ventricular CSF (no CP) and background was 12 and 7 (arb units) respectively. Figure 1D shows a map of T2_app_, generated from the multi-TE FLAIR acquisition where regions of high T2_app_ appear to be spatially co-localized to the CP within the lateral ventricles. This pilot study, therefore, suggested that the T2 of the signal co-localized to the choroid plexus from a multi-TE FLAIR sequence was markedly higher than brain tissue, an unexpected observation which to our knowledge has not been reported before.

### 3.2 Part ii) Performing bi-exponential fitting to understand more about the underpinnings of the long T2 component at the CP

**Fig. 2.**
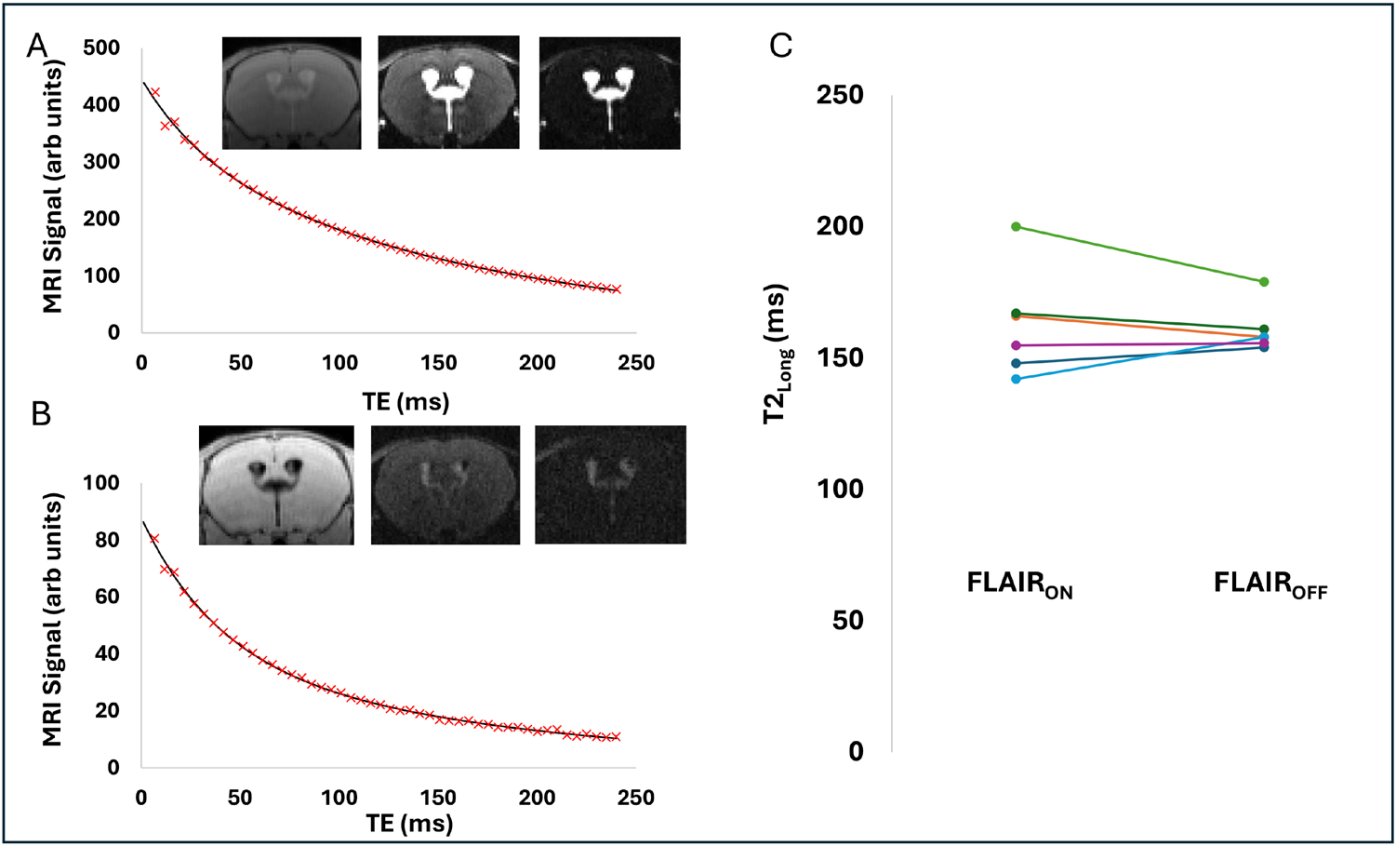
Bi-exponential fitting of the multi-TE FLAIR signal at the CP provides evidence for marked CSF signal contributions. (A) The mean signal from a ROI co-localised to the choroid plexus at increasing TE (red crosses) together with the 4-parameter bi-exponential fit (black line), taken from the multi-TE sequence without the FLAIR preparation [i.e. no inversion pulse]. Insert shows example images at increasing TE (NB images are scaled differently). (B) Same as (A), but with the FLAIR preparation from the same ROI as used in (A). (C) The estimated T2_Long_ from the bi-exponential fit with the FLAIR pulse on and off respectively. Each line represents an individual subject (n = 6).

We estimated the T2 of CSF by taking the slow decaying T2 component of the multi-TE acquisition (T2_long_) without the FLAIR preparation, where an excellent fit to the data was observed (Fig. 2A). The Akaike Information Criteria for the mono-exponential and bi-exponential model(s) were 105 ± 28 and 14 ± 26 respectively (p < 0.0001). We found a high degree of similarity between the T2_long_ estimates from an identical ROI with and without the FLAIR preparation: T2_long_ (FLAIR_OFF_) = 161 ms ± 9 ms; T2_long_ (FLAIR_ON_) = 163 ms ± 21 ms (Fig. 2C). The S0_long T2_ /[S0_short T2_ + S0_long T2_]ratio (FLAIR_ON_) was found to be 0.56 ± 0.08. Thus, these data lead us to surmise that the long T2_app_ of the signal co-localized to the CP from the multi-TE FLAIR acquisition (Fig. 1) is due to a significant proportion (56% ± 8%) of this signal deriving from CSF, given that the estimated T2_long_ (FLAIR_on_) matches closely to that of the T2_long_ (FLAIR_OFF_). The T2_Short_ estimates were 32.7 ms ± 5.2 ms (FLAIR_on_), 29.0 ms ± 3.5 ms (FLAIR_OFF_) respectively and were not significantly different. We propose that T2_short_ derives from the CP tissue given that the T2_short_ values are similar to those derived from recent work from Lee and colleagues (Lee et al., 2022).

**Fig. 3.**
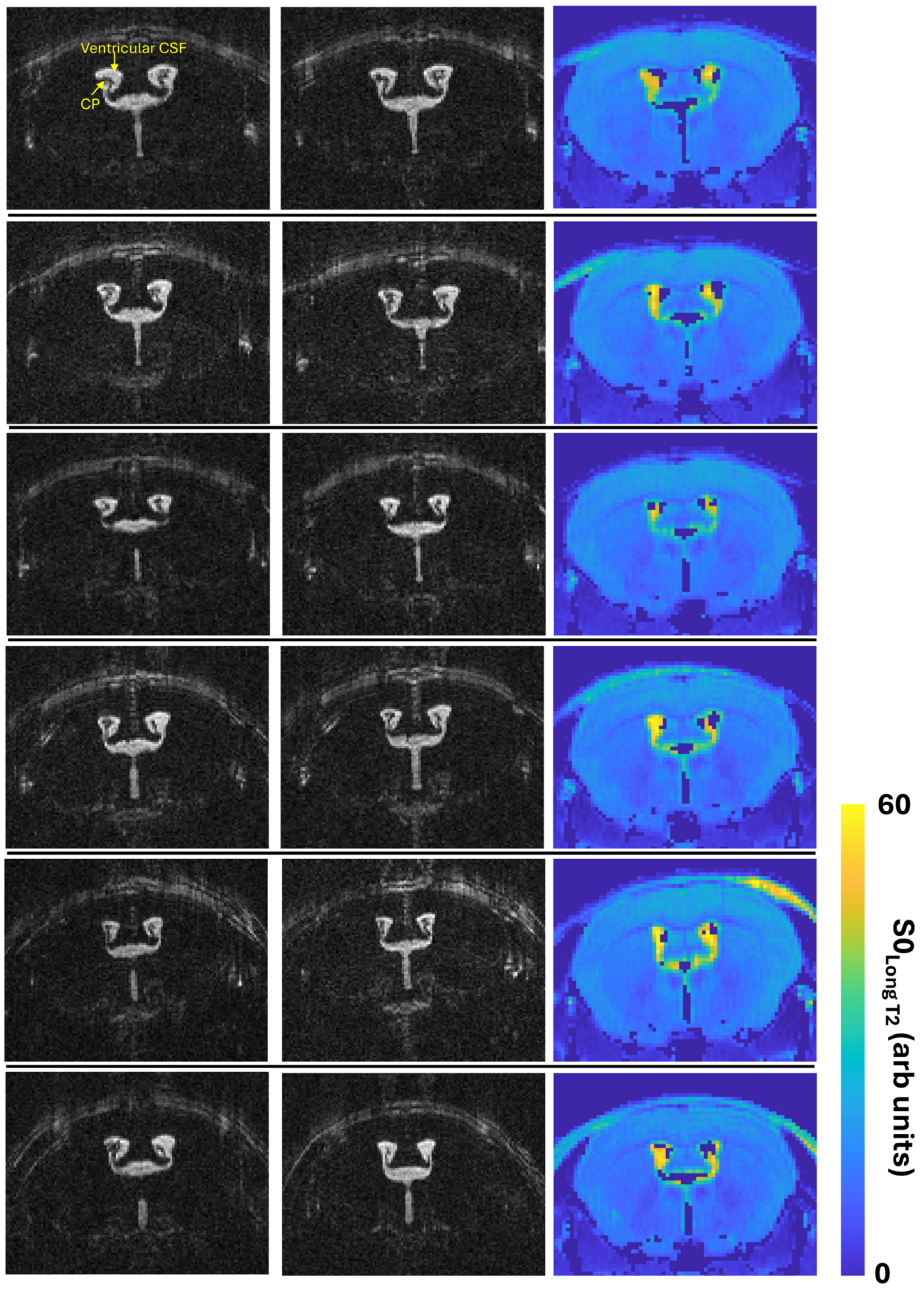
Maps of the signal contribution with a long T2 component (~160 ms) from the multi-TE FLAIR acquisition show high spatial colocalization to the choroid plexus in the lateral ventricles. Columns 1 and 2 show consecutive slices from the separate high resolution T2 weighted acquisition (designed to show the location of the CP within the lateral ventricles). Column 3 shows the maps of S0_Long T2_. Each row represents an individual mouse (n = 6).

The maps of the CSF signal contributions for each individual subject are shown in Figure 3 with comparison to the separate high-resolution T2-weighed scans (showing the location of the CP tissue within the lateral ventricles). Visual assessment of Figure 3 provides evidence that the regions of hyperintensity within these maps are specifically co-localized to the location of the CP tissue. Drawing an ROI at the ependymal ventricular wall returned a S0_long T2_ /[S0_short T2_ + S0_long T2_]ratio of 0.15 ± 0.06 which was markedly smaller than that from the CP ROI, 0.56 ± 0.08, p = 0.001. The T2_Long_ (FLAIR_off_) in a ventricular CSF ROI (no CP) was 188 (± 6) ms and was significantly greater (~15%) than the T2_Long_ from the CP ROI (FLAIR_OFF_).

### 3.3 Part iii) Investigating the effects of inversion pulse slice thickness on the multi-TE FLAIR measurements

Bi-exponential fitting of the signal at the CP (identical ROI for each mouse) based on multi-TE FLAIR images acquired with brain-wide and global inversion pulses returned highly similar CSF/CP tissue ratio measurements (see Supplementary Fig. S2). This suggests that, unlike the BCSFB-ASL technique (Evans et al., 2020), this phenomenon is minimally sensitive to perfusion of the CP. The S0_CSF_/[S0_CP_ + S0_CSF_] ratio measurements were 0.546 ± 0.07 and 0.564 ± 0.07 for the brain-wide and global inversion pulse respectively, which were also highly similar to that observed in part ii).

### 3.4 Part iv) Application of the Multi-TE FLAIR technique to the aged brain

**Fig. 4.**
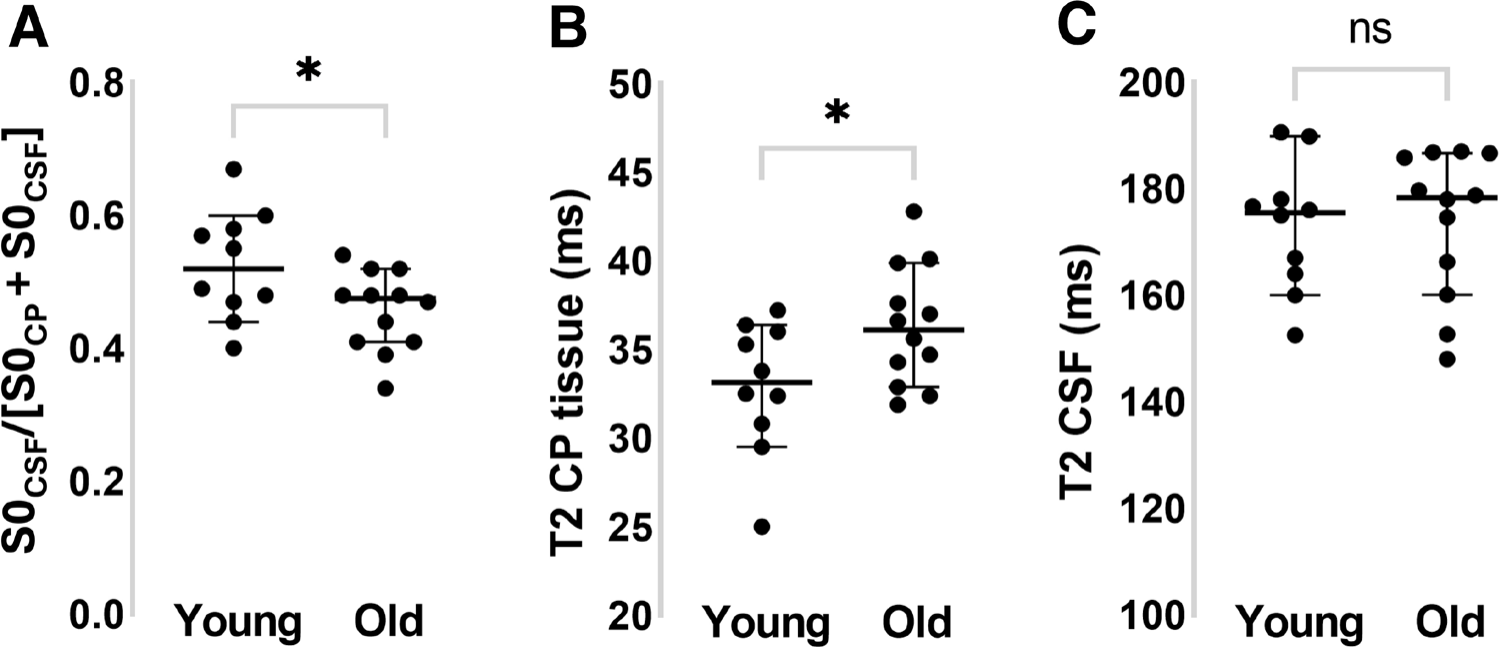
Investigating choroid plexus water exchange rate in the aged brain using multi-TE FLAIR. Output parameters from a bi-exponential fit to the signal in a CP ROI: (A) The S0_CSF_/[S0_CP_ + S0_CSF_] signal ratio within a CP ROI. (B) The T2 of the CP tissue. (C) The T2 of the CSF proximal to the CP For all plots (A-C), each dot represents an individual mouse (n = 10 young, n = 12 aged). *p < 0.05.

The results of the 4-parameter bi-exponential fitting to the signal co-localized to the CP for the aged and young control cohort are shown in Figure 4.

We record a significant decrease in the CSF/CP signal ratio (S0_CSF_/[S0_CP_ + S0_CSF_]) in the aged mouse brain as hypothesized, indicative of reduced water exchange rate at the CP in the aged brain (young: 0.53 ± 0.08; aged: 0.46 ± 0.06, p = 0.04). There was no difference in the T2 of the CSF component between the groups (young: 173 ± 12 ms; aged: 174 ± 14 ms, p = 0.89) with a significant increase in the estimated T2 of the CP tissue in the aged vs. young cohort (young: 33 ± 4 ms; aged: 36 ± 3 ms, p = 0.04). The TI_null_, which was calculated for each mouse individually based on a pre-scan at multiple TIs, was highly similar between the young and aged group (young: 2132 ± 45 ms; aged: 2139 ± 29 ms, p = 0.64) suggesting that the T1 of the ventricular CSF is also similar between the groups.

### 3.5 Part v) Preliminary application of the multi-TE FLAIR measurement to the human brain at 3T

**Fig. 5.**
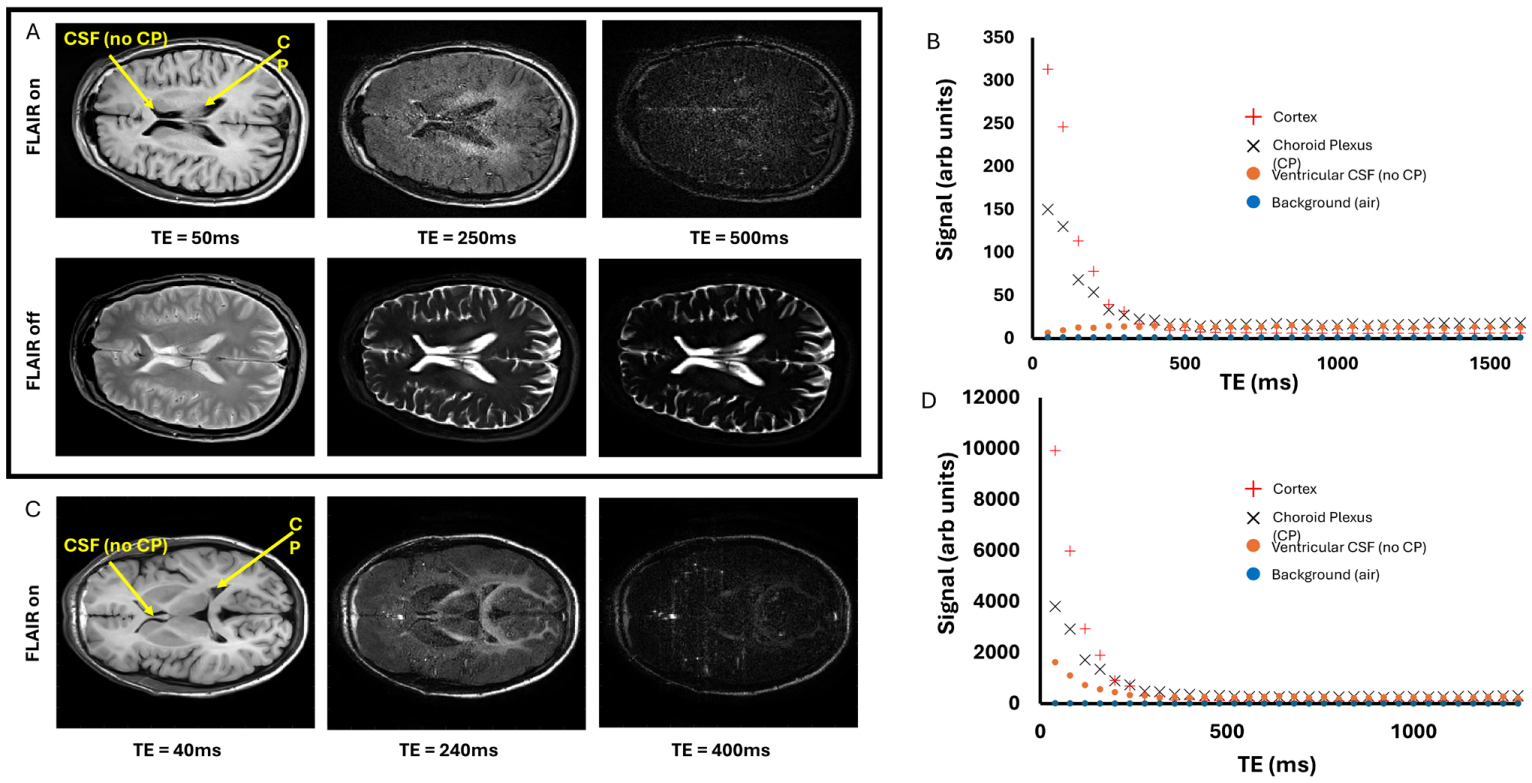
Pilot Studies of the Multi-TE FLAIR Technique at 3T. (A) Axial images at TEs of 50, 250 and 500 ms with the FLAIR prep on (top row) and off (bottom row) from a healthy volunteer on a Siemens 3T system. (B) The mean signal within the indicated ROIs (insert) as a function of TE for the data with the FLAIR prep on acquired on a Siemens 3T system. (C) Axial images at TEs of 40, 240 and 400 ms with the FLAIR prep on from a healthy volunteer on a Philips 3T system. (D) The mean signal within the indicated ROIs (insert) as a function of TE for the data with the FLAIR prep on acquired on a Philips 3T system.

Figure 5A shows example axial images at variable echo times with FLAIR pulse on (top row) and off (bottom row) respectively acquired in the human brain on a 3T Siemens system. Visual assessment of the images at long TE with the FLAIR pulse on, shows little evidence for hyperintensity at the CP, in contradiction to our findings in the mouse brain (see Fig. 1A for example). Accordingly, plotting the signal as a function of TE at ROIs in the CP and lateral ventricles (no CP) returned no clear evidence for greater long-T2 signal contributions (Fig. 5B). Images without the FLAIR prep, however, indicate that the identical multi-TE acquisition can successfully capture the long-T2 behavior of the CSF signal when it has not been nulled by the prior inversion pulse (Fig. 5A bottom row). Highly similar findings were observed in the healthy volunteer imaged on the Philips 3T system (Fig. 5C, D). Together, therefore, these pilot studies indicate that the same phenomenon reported in parts i-iv) in the mouse brain at 9.4T above cannot be captured with equivalent sensitivity in the human brain at 3T.

## 4 Discussion

In this work, we introduce the concept of a multi-TE FLAIR MRI acquisition to non-invasively image the exchange of water between the choroid plexus and proximal CSF. This measurement may represent a novel and non-invasive biomarker of choroid plexus derangement associated with pathology. Moreover, this novel contrast mechanism appears to be highly spatially specific to the choroid plexus, meaning that it could be a useful method for accurately pinpointing the location of the choroid plexus and perhaps for accurately estimating its volume (Li et al., 2025). Applying the method to the aged brain, we detect a reduction in the estimated rate of CP-CSF water exchange. However, pilot experiments in the human brain suggest that the sensitivity of the method is markedly reduced when applied in humans at 3T.

This work originated with a pilot study that yielded an unexpected finding: the high T2 of the signal spatially co-localized to the CP in a multi-TE FLAIR acquisition, despite evidence that the CSF in the ventricles (with no CP tissue) was successfully nulled [see Fig. 1]. We then set about trying to understand more about the compartmental origins of this high T2 signal component, and to investigate whether it derived from the stromal tissue of the CP (for example) or somehow from CSF, despite the FLAIR preparation. To investigate the latter explanation (i.e., whether a significant source of the long-T2 signal derived from water molecules in the CSF), we fitted the multi-TE FLAIR signal at the CP to a 4-parameter unconstrained biexponential model to estimate the T2 value of the ‘slow-decaying’ transverse component. It was observed that this matched the T2 of the CSF (estimated using an identical acquisition and analysis but without the FLAIR preparation; see Fig. 2), providing strong evidence that the source of the high T2 value does, indeed, derive from water in the CSF. Indeed, this CSF-derived signal appears to account for ~56% of the total, with the rest deriving from the CP tissue itself (lower T2). The clear spatial co-localization of this CSF-derived signal to the CP (Fig. 3) provides evidence that this phenomenon is specific to the CP, which we surmise to reflect high rates of water exchange between the CP and CSF during the TI, primarily due to the large surface area of the CP (arising in part due to dense micro villi). To clarify further, we propose that the rapid exchange of water between the CSF and the CP tissue means that during the TI (~2.1 s) a sizable fraction of the water molecules spend time within the microenvironment of the CP tissue which increases their rate of longitudinal magnetization recovery (compared to the CSF) but at the time of measurement they have crossed into the CSF (with its characteristic long T2 relaxation time). Of note, we think that the high-T2 component is not likely to derive from blood, as both in-vitro and in-vivo measures of blood T2 return a range of 10–40 ms at 9.4T (Lee et al., 1999; Ohene et al., 2019; Wells et al., 2013). It is important to note, however, that our observations could also be explained by the existence of a specific water compartment in the CP that has a T2 highly similar to that of CSF but with a markedly different T1. Given the rationale described above, however, we believe that water exchange is the dominant source of contrast at play.

What was particularly notable about the multi-TE FLAIR measurements at the CP was the marked contribution of water molecules in the CSF to the measured signal: 56% was estimated to be in the CSF (long T2 value) and 44% in the CP tissue (short T2 value). This marked CSF signal contribution can be seen visually (e.g. Fig. 1A) where the CP clearly ‘lights’ up at longer TEs. From a methodological perspective, this relatively equal balance of signal contributions with short and long T2 is beneficial to precise bi-exponential fitting of the MRI signal, where the model assumes that there is exchange during the TI but no exchange (or slow exchange limit) during the TEs. Physiologically, we speculate that the marked movement of the CP during cardiac and respiratory cycles, together with the high apparent diffusion coefficient (ADC) of the CSF and the large surface area of the CP tissue, combine to give this interface a remarkably high capacity for CSF-CP tissue water exchange. Interestingly, the slow decaying T2 value of the ventricular CSF (FLAIR_OFF_) was found to be subtly greater than that of the slow decaying T2 value taken from the CP ROI (FLAIR_OFF_). This may reflect a combined effect of CP water exchange during the TE and differences in the composition of the CSF as concluded by Jiang and colleagues (Jiang et al., 2023) in their recent study that mapped the T2 of the CSF in the human brain.

The contrast mechanisms employed here by the multi-TE FLAIR method share several features with the T1-PALAN technique, and to a lesser extent the MISL method (recently introduced by Li and colleagues, designed to measure water exchange between tissue and CSF (Li & Xu, 2022; Li et al., 2022). However, the data in the present study appear to yield maps of tissue-to-CSF water exchange that are tightly spatially co-localized to the CP whereas the maps of water exchange presented in these earlier studies appear to be more diffuse within the ventricular compartments. This suggests that the present multi-TE FLAIR method can capture signal that is specific to water exchange at the CP whereas the T1-PALAN method appears to detect alternative water-exchange phenomena (see for example Fig. 4 in Li et al. (2022) where the maps of water exchange appear hyperintense at the edges of the ventricles, in turn implying sensitivity to exchange across the epidymal layer). Indeed, when we examined the signal from an ROI at the ependymal layer at the edges of the ventricles, the average CSF contribution to the signal was only 15% which likely reflects both an imperfect choice of TI for successful nulling of free CSF and perhaps a degree of water exchange between the CSF and ependymal layer. The precise reasons for this difference in the spatial distribution of exchange are unknown but encouragingly this suggests that the methods are sensitive to different sites of exchange and therefore could provide distinct and non-invasive insight into the compartmental water exchange in the brain. An alternative method for imaging water exchange at the choroid plexus using similar T2-based contrast mechanism was recently introduced (Wu et al., 2024). Encouragingly they showed that their measure of CP water exchange was sensitive to acetazolamide in the rat brain, suggesting that such estimates of water exchange may correlate to changes in CSF secretion. Future work will examine the sensitivity of the current multi-TE FLAIR measurement to pharmacological modulation of CSF secretion.

Here, we used an inversion pulse with a slice thickness that was sufficient to cover the entire head of the mouse. This was to ensure that the measurements were not sensitive to any CSF inflow effects. It is important to note, however, that the measurements have limited sensitivity to the delivery of labelled blood-water across the CP to the CSF, a technique that we previously introduced to probe the function of the BCSFB, that is highly sensitive to CP perfusion (Evans et al., 2020). This can be seen in Supplementary Figure S2 where the S0_CSF_/[S0_CP_ + S0_CSF_] ratio, calculated from the bi-exponential fit to the multi-TE FLAIR data, had minimal sensitivity to a global vs. brain-selective inversion pulse. This provides strong evidence that the method introduced here reflects a distinct physiological phenomenon to the BCSFB-ASL technique, which we surmise to be the rapid exchange of water molecules between the CSF and CP tissue as opposed to the BCSFB-mediated delivery of labeled blood water to the CSF.

When equivalent multi-TE FLAIR measurements were made in the human brain, the sensitivity of the method appeared to be much reduced relative to the mouse brain data. This can be seen by visual comparison of the long-TE FLAIR images in Figure 1 and Figure 5 where the signal from the CP specifically ‘lights up’ in the mouse brain but is not visible in the human brain. We conclude, therefore, that the rate of CP-CSF water exchange is markedly greater in the mouse brain compared to the human brain, which thus reduces the proportion of long T2 signal at the CP at the time of the multi-TE readout. We surmise that this is due to the following: here, we are proposing that the high S0_LongT2_ that overlays with the CP in the multi-TE FLAIR images in the mouse brain is reflective of rapid exchange of water molecules between the CSF and CP tissue during the TI. This rapid exchange occurs because the micro-villi of the CP impart a large surface area to interface the two compartments. In addition, the CP (and surrounding CSF) undergoes considerable movement with cardiac and respiratory pulsation which likely plays an important role to facilitate rapid mixing/exchange of water molecules between the CP tissue and CSF during the TI. The mouse heart rate is ~480 beats per minute and respiration rate ~120 breaths per minute under isoflurane anaesthesia. Given the TI of ~2.1 s in the mouse brain at 9.4 T and ~1.4 s in the human brain at 3 T, this means that there are ~17 heart beats and 4 breaths in the mouse brain during the TI but only ~1–2 heart beats and ~0–1 breaths in the human brain during the TI. This is likely to markedly reduce the degree of pulsatile-driven mixing/exchange of water between the CP and CSF that occurs during the TI in the human brain relative to the mouse brain.

Interestingly, however, visual inspection of some previously acquired FLAIR images of the human brain at extended echo times in the literature does display hyperintensity at the CP, indicating the presence of a long-T2 CSF signal contribution as characterized here in the mouse brain. For example, Eichinger and colleagues used a double-inversion FLAIR sequence with a TE of 328 ms at 3 T which appear to capture hyperintensity at the CP (e.g., Fig. 1a in (Eichinger et al., 2017)). This may be due to the increase in the effective TI (2550 ms), allowing for greater CP-CSF water exchange. As another example, Visser et al. implement a magnetization-prepared FLAIR readout at 7 T to increase the T2 weighting of the image which also displays hyperintensity at the CP (e.g., Fig. 5 in Visser et al. (2010)). Recently, a novel MRI method has been developed that appears to image CP water exchange with high sensitivity and at high spatial resolution which may, therefore, represent a better method for imaging water exchange at the CP in the human brain (Taso & Alsop, 2025). The present multi-TE FLAIR method may offer a useful and easily implementable tool for measuring water exchange at the CP in pre-clinical research studies, knowing that similar measurements can be applied clinically using the approach by Taso and Alsop, for example (Taso & Alsop, 2025).

An important aspect of the method is that it is highly sensitive to the efficiency of ventricular CSF nulling due to the FLAIR image preparation. Ideally ‘free’ CSF in the ventricles (i.e., not spatially co-localized to the CP) should be perfectly nulled at the chosen TI but, in practice, this is not always practically achievable. When generating maps, such as those shown in Figure 3, it is important to ensure that the resultant maps are not an artifact of thresholding based on voxel intensity whereby voxels are included that contain CP tissue and excluded in free CSF. This could potentially mean that the maps of S0_LongT2_ do not reflect exchange of water between the tissue/CSF but rather imperfect ventricular CSF nulling combined with voxel-intensity based thresholding. However, as shown in Figure 1, we were able to achieve good nulling of ventricular CSF and found that the CSF contribution in an ependymal ROI (at the edge of the ventricle) was markedly less than at the CP (0.15 vs. 0.56). In addition, we performed an independent optimization of the TI_null_ for each individual mouse in the aged vs. young cohort to control for any possible differences in the T1 of the CSF between young and aged mice. Furthermore, irrespective of the accuracy of ventricular CSF nulling, it is important to note that the method will be sensitive to the TI as this will determine the time for water exchange. Furthermore, the S0_CSF_/S0_CP_ ratio will depend on the T1 values of the CP and the CSF proximal to the CP which is unknown here (although the TI_null_ of the free CSF was very similar between the young and old cohort). Here, we report the ratio S0_CSF_/[S0_CP_ + S0_CSF_] as a measure of water exchange rate between the CP tissue and proximal CSF. The primary reason that we report this ratio rather than just S0_CSF_, for example, is that it accounts for the variable degree of CP tissue within the ROI. This will be inherently variable given the inevitable partial volume effects of the CP and CSF that occurs with in-vivo MRI assessment of the CP together with the marked between-subject variability of its location within the lateral ventricle system as shown in Figure 3 (columns 1–2). Therefore, we report the ratio S0_CSF_/[S0_CP_ + S0_CSF_] to control for a scenario where the estimated S0_CSF_ is high, not because of rapid water exchange, but because there is a large amount of CP tissue within the ROI. So, for example, if twice as much CP tissue is present in a voxel, we would expect twice as much water that has undergone exchange with the CP and is present in the CSF at the time of measurement (i.e., S0_longT2_) for the same exchange rate. It is also important to note that the multi-TE acquisition employed here will be sensitive to B1 and B0 inhomogeneities and these should be minimized for precise T2 estimation. Here, we recorded a significantly lower T2 of the CP tissue in the old vs. young brain (see Fig. 4B). Given that changes in T1 and T2 tend to be monotonically correlated, this may suggest that the T1 of the CP tissue is also decreased (though we did not seek to directly measure this in the present study). If this were the case, this may act to artificially increase the S0_CSF_/[S0_CSF_ + S0_CP_] ratio and hence the inferred rate of CP-CSF water exchange. The calculated S0_short T2_ could potentially be used to estimate the volume of the CP tissue, although a higher resolution and multiple slice readout would benefit this particular measurement. In addition, S0_short T2_ will be sensitive to the T1 of the CP tissue and because it is just a signal intensity, it would need to be corrected for surface coil sensitivity, for example in order to achieve reliable comparisons between different subjects.

Here, we found a reduction in our measure of CP-CSF water exchange rate in the aged mouse brain. This may reflect the reduction in microvilli length that has recently been reported in the aged mouse brain (Scarpetta et al., 2023) and the resultant reduction in surface area interfacing the two compartments. Interestingly, we observed a significant increase in T2_CP_ in the aged mouse brain compared to the young controls, although we do not know the biological changes underpinning this observation at this moment in time.

To conclude, here we provide evidence that a multi-TE FLAIR acquisition can yield semi-quantitative maps of CP to CSF water exchange rate with high sensitivity in the mouse brain at 9.4 T. The non-invasive technique may be useful as a research tool to better understand the contribution of the CP to pathology as well as to help unlock its potential as a novel therapeutic target.

## Supplementary Material

Supplementary Material

## Data Availability

Upon publication, the MRI data will be made freely available for download at https://rdr.ucl.ac.uk/authors/Jack_Wells/6768476.
