# Supplementary material for: Non-invasive MRI of choroid plexus-cerebrospinal fluid water exchange using multi-TE FLAIR
Source: Imaging Neurosci (Camb). 2026 Jan 16;4:IMAG.a.1097. doi: 10.1162/IMAG.a.1097 (PMC12813853; doi:10.1162/IMAG.a.1097)
Supplement: Supplementary Material [file IMAG.a.1097_supp.pdf]

Supplementary Material

**Notation and Metrics**

Please find below a summary of the notation and metrics used in this work:

$T2_{long}$ : the estimated T2 of the slow decaying component from bi-exponential fitting of the signal captured with the multi-TE readout taken from a ROI overlayed on the CP.

$T2_{short}$ : the estimated T2 of the fast decaying component from bi-exponential fitting of the signal captured with the multi-TE readout taken from a ROI overlayed on the CP.

$S0_{long\ T2}$ : the signal contribution of the long T2 component from bi-exponential fitting of the signal captured with the multi-TE readout taken from a ROI overlayed on the CP.

$S0_{short\ T2}$ : the signal contribution of the short T2 component from bi-exponential fitting of the signal captured with the multi-TE readout taken from a ROI overlayed on the CP.

$S0_{long\ T2}/[S0_{long\ T2} + S0_{short\ T2}]$ : the fractional signal contribution with a long T2 component to the total measured signal, taken from a ROI overlayed on the CP.

$S0_{CP}$ : the signal contribution from the CP tissue from bi-exponential fitting of the signal captured with the multi-TE readout taken from a ROI overlayed on the CP. This is the same as $S0_{short\ T2}$ but is assigned as $S0_{CP}$ after we had evidence that this component originated from the CP tissue.

$S0_{CSF}$ : the signal contribution from the CSF from bi-exponential fitting of the signal captured with the multi-TE readout taken from a ROI overlayed on the CP. This is the same as $S0_{long\ T2}$ but is assigned as $S0_{CSF}$ after we had evidence that this component originated from the CSF.

$S0_{CSF}/[S0_{CSF} + S0_{CP}]$: the fractional signal contribution from the CSF to the total measured signal, taken from a ROI overlayed on the CP. This represents our proposed estimate of CP-CSF water exchange rate.

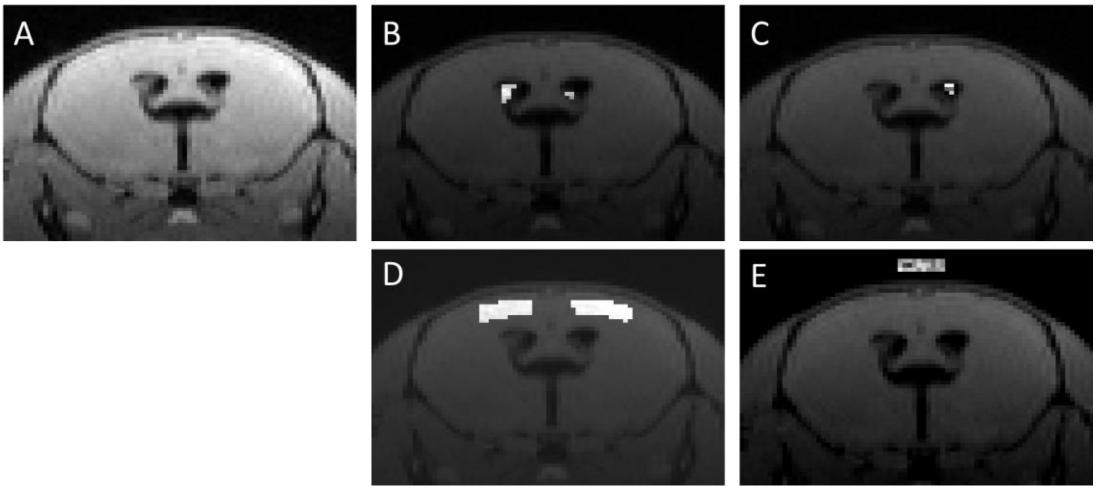


**Supplementary Figure 1**: A. *Example image acquired at the earliest TE ($FLAIR_{ON}$). Example of resultant CP ROI (B), Ventricular CSF (no CP) ROI (C), Cortical ROI (D) and background (air) ROI (E).*

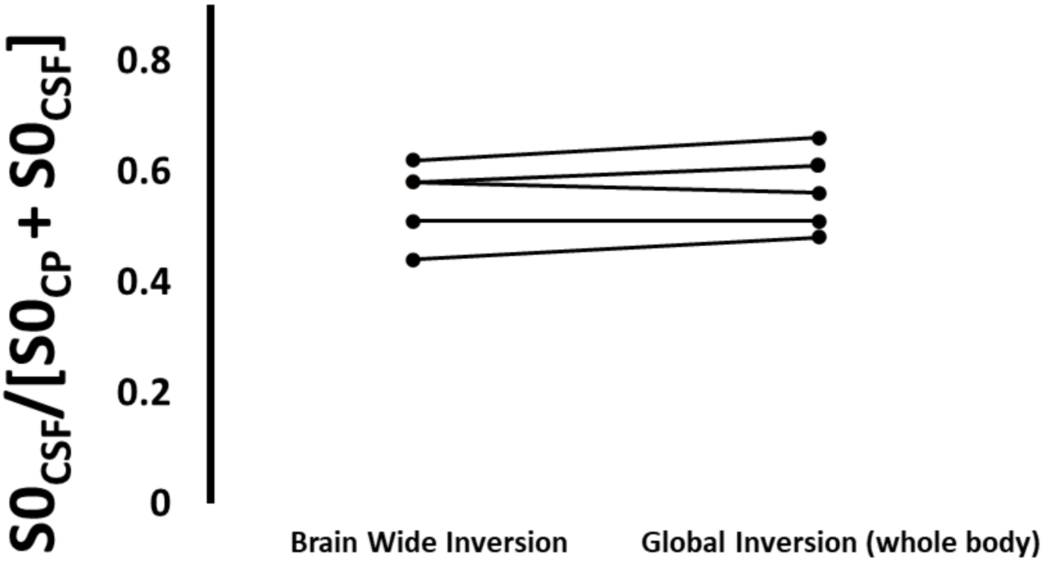


**Supplementary Figure 2: The Multi-TE FLAIR Measurement at the CP has Negligible Dependence on Blood-to-CSF Labelled Water Transfer.** *The estimated CSF/CP Signal ratio with a brain wide and global (whole body) FLAIR inversion pulse. Each individual line represents each individual mouse (n = 5).*
